# Viral and Atypical Bacterial Detection in Acute Respiratory Infection in Children Under Five Years

**DOI:** 10.1371/journal.pone.0018928

**Published:** 2011-04-18

**Authors:** Patrícia G. M. Bezerra, Murilo C. A. Britto, Jailson B. Correia, Maria do Carmo M. B. Duarte, Angela M. Fonceca, Katie Rose, Mark J. Hopkins, Luis E. Cuevas, Paul S. McNamara

**Affiliations:** 1 Instituto de Medicina Integral Professor Fernando Figueira (IMIP), Recife, Brazil; 2 Faculdade Pernambucana de Saúde, Recife, Brazil; 3 Faculdade de Ciências Médicas, Universidade de Pernambuco, Recife, Brazil; 4 Department of Child Health, University of Liverpool, Institute of Child Health, Alder Hey Children's Hospital, Liverpool, United Kingdom; 5 Liverpool Specialist Virology Centre, Royal Liverpool University Hospital, Liverpool, United Kingdom; 6 Liverpool School of Tropical Medicine, Pembroke Place, Liverpool, United Kingdom; University of Georgia, United States of America

## Abstract

**Background:**

Acute respiratory infection (ARI) is a leading cause of morbidity and mortality in children worldwide. This study aimed to determine the viral and atypical bacterial causes of different severities and clinical manifestations of ARI in preschool children from low-income families in North-East Brazil.

**Methods:**

Clinical/demographic data and nasopharyngeal aspirates (NPA) were prospectively collected from children <5 years presenting with ARI over one year to a paediatric A&E department. Disease severity was grouped according to presence of lower respiratory tract signs, need for hospital admission and need for oxygen. Clinical manifestation of ARI was based on discharge diagnosis from hospital with four conditions predominating: bronchiolitis, pneumonia, episodic viral wheeze/asthma and upper respiratory tract infection. Multiplex PCR was used to detect 17 common respiratory viral and atypical bacterial pathogens in NPA.

**Findings:**

407 children with a median age of eight months were recruited. Pathogens were detected in 85·5% samples with co-infection being particularly common (39·5%). Respiratory Syncytial Virus (RSV; 37%), Adenoviruses (AdV; 25%), Rhinoviruses (hRV; 19%), Bocavirus (hBoV; 19%), human Meta-pneumovirus (hMPV; 10%) and *Mycoplasma pneumoniae (Mpp;* 10%) were most prevalent. Detection and co-infection rates were similar in all severities and clinical manifestations of ARI apart from RSV, which was associated with more severe disease and specifically more severe cases of bronchiolitis, and *Mpp*, which was associated with more severe cases of pneumonia. *Mpp* was detected in 17% of children admitted to hospital with pneumonia.

**Interpretation:**

This study underlines the importance of viral and atypical bacterial pathogens in ARI in pre-school children and highlights the complex epidemiology of these pathogens in this age group. Generally, viruses and atypical bacteria were detected in all severities and clinical manifestations of ARI but RSV and *Mpp* were associated with more severe cases of bronchiolitis and pneumonia respectively.

## Introduction

Acute respiratory infection (ARI) is an important cause of morbidity and mortality with a worldwide disease burden estimated at 112 900 000 disability adjusted life years (DALYs) and 3·5 million deaths.[1], [2] A strict definition of ARI would include all infections of the respiratory tract. However, in practice, acute lower respiratory infection accounts for most of the serious disease burden. ARI causes about 20% of all deaths in pre-school children worldwide, with 90% of these deaths being due to pneumonia. Risk factors for severe ARI include malnutrition, low birth weight, passive smoking, non-breastfeeding, low socio-economic status, overcrowding, immunodeficiency and HIV infection, and consequently, most of the morbidity associated with ARI is found in the developing world.[3], [4], [5]

ARI causative organisms are predominantly bacterial (most commonly *Streptococcus pneumoniae* and *Haemophilus influenzae*) or viral, although it is not possible to differentiate between them based on clinical signs or radiology. New pathogens are frequently being reported in the literature, including Coronaviruses (NL63 and HKU1), human Metapneumovirus (hMPV) and human Bocavirus (hBoV).[6], [7], [8], [9] This expansion in the number of pathogens, combined with an increased ability to simultaneously test for multiple organisms, has highlighted the potential role of co-infection, with the detection of more than one pathogen from a single sample, although their significance with regard to disease severity remains debatable.[10], [11]

The causes of ARI in children under-5 years have been studied in different environments and populations. Respiratory syncytial virus (RSV), hMPV, Rhinoviruses (hRV) and Para-influenza viruses (PIV) have consistently been shown to predominate, with some displaying strong seasonal peaks and with co-infection with more than one viral pathogen occurring in 4–33% of children.[11], [12], [13], [14] Few studies however have attempted to determine whether particular viruses are associated with differing severities of disease. Those that have, have concentrated on the more severe end of the disease severity spectrum i.e. those children with RSV disease hospitalized or ventilated on intensive care.[15] Similarly, few studies have examined in detail the viral causes of specific clinical conditions in the under-5 age group.[16]

The aim of this study was to determine the prevalence and seasonal distribution of viral and atypical bacterial pathogens in a cohort of well-characterised children under the age of 5 years presenting to a paediatric A&E department in North-Eastern Brazil with various severities and clinical manifestations of ARI.

## Methods

### Ethics Statement

The Ethics Committee at IMIP and the National Research Ethics Office of Brazil approved the study and written informed consent was obtained from the parents prior to enrolment.

### Setting and study design

This prospective cross-sectional study of children with ARI was conducted between April 2008 and March 2009 at the Instituto de Medicina Integral Prof Fernando Figueira (IMIP), a large publically funded teaching hospital in Recife, Pernambuco, North-East Brazil. Between one third and one half of the population of Recife lives in poverty in urban slums and IMIP's paediatric A&E department primarily provides primary, secondary and tertiary medical care to these low-income families. HIV prevalence in Brazil and Recife is low.

After conducting a pilot study to examine feasibility (specifically aspects of accrual, data and sample collection), a trained research assistant approached consecutive patients with ARI between 7 am and 3 pm (Monday to Friday) whilst they were in the paediatric A&E department and collected clinical and demographic data from parents/guardians.

### Subjects

All children aged less than five years with upper and/or lower respiratory tract manifestations of ARI of less than 7 days duration were eligible for inclusion. Baseline observations including temperature, oxygen saturation, respiratory and pulse rates were recorded at the time of consulting the A&E department and daily (if admitted) thereafter in all participants. Nasopharyngeal aspirate (NPA) collection was performed by a research assistant within four hours of consultation using a standardised protocol.[17]

### Disease severity

Disease severity was assessed at the time of enrolment and categorized as follows:

1. *Very mild (*upper respiratory tract symptoms/signs only)
2. *Mild* (lower respiratory tract symptoms/signs +/− upper respiratory tract symptoms/signs but not needing hospital admission)
3. *Moderate* (lower respiratory tract symptoms/signs +/− upper respiratory tract symptoms/signs, needing hospital admission but with oxygen saturations in air >93% on pulse oximetry)
4. *Severe* (lower respiratory tract symptoms/signs +/− upper respiratory tract symptoms/signs, needing hospital admission and oxygen with saturations in air <93%)

### Clinical manifestations of ARI

The clinical outcome and diagnosis for each child were recorded following discharge from hospital. The discharge diagnosis for each child was made by attending physicians not involved in the study and based on standard clinical criteria. Thus bronchiolitis was diagnosed in children <18 months in whom upper respiratory symptoms preceded lower respiratory symptoms of wheeze, tachypnoea and signs of respiratory distress. Pneumonia was diagnosed in children with fever, tachypnoea and respiratory distress where focal or diffuse crackles or decreased vesicular sounds were present on auscultation. Most diagnoses of pneumonia were based on clinical criteria alone, but radiographic findings were used in some. A diagnosis of episodic viral wheeze (EVW)/asthma was made in children in whom discreet episodes of wheeze occurred, often in association with a presumed viral upper respiratory tract infection. Upper respiratory tract infections (including croup) were diagnosed based on symptoms such as coryza, earache, sore throat and stridor.

### Nucleic acid extraction

Each NPA was diluted in 3 mls of normal saline before being centrifuged at 500 g for 10 minutes at 4°C. The resultant cell pellet was resuspended in a solution of 2-mercaptoethanol (Sigma – Aldrich, Brazil) and RLT lysis buffer (QIAGEN, Crawley, UK) and then frozen, along with the supernatant, at −70°C. The time from sample collection to freezing was less than four hours in all cases and the longest that any sample was kept at −70°C prior to nucleic acid extraction was one year.

DNA and RNA were co-extracted from 200 µl sample supernatant using the QIAsymphony Virus/Bacteria Mini Kit (Pathogen Complex 200 protocol) according to manufacturers instructions (QIAGEN, Crawley, UK) prior to PCR analysis.

### PCR assays for respiratory pathogens

Seventeen viral and atypical bacterial respiratory pathogens were included in the panel. Multiplex reverse-transcription PCR was performed as described previously for the detection of RSV, hMPV, Influenza viruses A & B (Flu A&B), PIV 1-4, and hRV.[18] Additional primer-probe sets were utilised for detection of CoVs (OC43, NL63, 229E, HKU1), hBoV, Adenovirus (Adv), *Mycoplasma pneumoniae* (*Mpp)* and *Chlamydia pneumoniae* (*Cpp)* as shown in **Table 1**. Assays with RNA targets were carried out under conditions described by Hopkins et al.[18] PCRs with DNA targets utilised 10 ul purified nucleic acid from the Qiasymphony with the Roche LC480 Probes Master kit (Roche Diagnostics, Burgess Hill, UK) or Qiagen Quantitect Probe PCR kit (Qiagen, Crawley, UK) for the AdV, *Mpp*, *Cpp* multiplex PCR and hBoV PCR, respectively. Thermal cycling conditions were as described previously,[18] except for removal of the 50°C hold for reverse transcription, and the enzyme activation hold at 95°C was extended to five minutes for the Roche LC 480 Probe Master kit or fifteen minutes for the Qiagen Quantitect Probe PCR kit. All assays were performed in a Lightcycler 480 real-time PCR machine (Roche Diagnostics, Burgess Hill, UK).

**Table 1:** Additional oligonucleotide primers and probes used in this study.

Assay	Oligonucleotide	Sequence (5′ to 3′)	Concentration (µM)	Citation
CoV-4plx	OC43 F	CGATGAGGCTATTCCGACTAGGT	0.4	[32]
	OC43 R	CCTTCCTGAGCCTTCAATATAGTAACC	0.4	
	OC43	(Cyan500)– TCCGCCTGGCACGGTACTCCCT–(BHQ1)	0.16	
	NL63 F	ACGTACTTCTATTATGAAGCATGATATTAA	0.4	[32]
	NL63 R	AGCAGATCTAATGTTATACTTAAAACTACG	0.4	
	NL63	(FAM)– ATTGCCAAGGCTCCTAAACGTACAGGTGTT –(BHQ1)	0.16	
	229E F	CAGTCAAATGGGCTGATGCA	0.4	[32]
	229E R	AAAGGGCTATAAAGAGAATAAGGTATTCT	0.4	
	229E	(HEX)– CCCTGACGACCACGTTGTGGTTCA –(BHQ1)	0.16	
	HKU1 F	TTACTTTCCACACTTTTCATCTCTCTG	0.4	
	HKU1 R	CGGAAGCAGCCAACGAAATTC	0.4	
	HKU1	(LC640)– CGCCCACTTGAAGCCGAGACCG –(BHQ2)	0.16	
CMA-3plx	Cpp F	CAAGGGCTATAAAGGCGTTGCT	0.2	[27]
	Cpp R	ATGGTCGCAGACTTTGTTCCA	0.2	
	Cpp	(LC670)– TCCCCTTGCCAACAGACGCTGG –(BHQ2)	0.1	
	Mpp F	GGAATCCCAATGCACAAGAACA	0.4	[27]
	Mpp R	GCTTTGGTCAACACATCAACCTT	0.4	
	Mpp	(LC610)– GCCTTGAAGGCTGGGTTTGCGCTA –(BHQ2)	0.1	
	Adv F	GCC ACG GTG GGG TTT CTA AAC TT	0.4	[33]
	Adv R	GCC CCA GTG GTC TTA CAT GCA CAT C	0.4	
	Adv	(FAM)– TGCACCAGACCCGGGCTCAGGTACTCCGA –(BHQ1)	0.2	
hBoV 2plx	hBoV NS F	CTTGGGCGGGACAGAATGC	0.4	
	hBoV NS R	AACAGAATTGCCACCAACAACC	0.4	
	hBoV NS	(FAM)– TCAAGCATAGAGACAGT –(MGB)	0.2	
	hBoV NP F	GCTCGGGCTCATATCATCAGG	0.4	
	hBoV NP R	CTCCCTCGTCTTCATCACTTGG	0.4	
	hBoV NP	(VIC)– AAT CAG CCA CCT ATC –(MGB)	0.2	

All oligonucleotides were purchased from Metabion Ltd, Germany, except MGB probes which were obtained from Applied Biosystems, Warrington, UK.

### Statistical Analysis

Differences in pathogen prevalence between disease severity categories or different clinical manifestations were examined using a Fisher Exact test, considering a p value <0·05 as significant. Statistical calculations were performed using SPSS 18·0·1 statistical package (SPSS Inc, Chicago).

For the purposes of analysis the following pathogens were grouped together: CoV OC43, CoV HKU1, CoV 229E and CoV NL63 (CoV); Influenza A and Influenza B (Flu); and PIV 1-4 (PIV).

## Results

### Demographic and Clinical Characteristics

407 children (236 males) under five years of age attending the A&E department were recruited. Their median (range) age was 8 (0–57) months, with 69% of participants being <12 months of age. Nineteen (4.7%) children had other significant co-morbidities and 92% had been or were still being breastfed. Overall, 43% of the children came from households with at least one smoker. The mean (±SD) household income was US$275 (±160), which is approximately half the average monthly income in Brazil and 43% of mothers and 44% of fathers had completed the national compulsory nine years education. The mean (±SD) number of people sharing the bedroom with the child was 3·0 (±1·6), with 26% sharing with at least four people.

Bronchiolitis, pneumonia, EVW/asthma and upper respiratory tract infections (URTI) comprised the majority of discharge diagnoses. Half (52%) of the children were admitted to the hospital wards and three died. Half of the children with bronchiolitis (105/211; 49·8%), 77.1% with pneumonia (84/109) and 23.6% with EVW/asthma (13/55) were hospitalised.

### PCR Results

**Figure 1:**
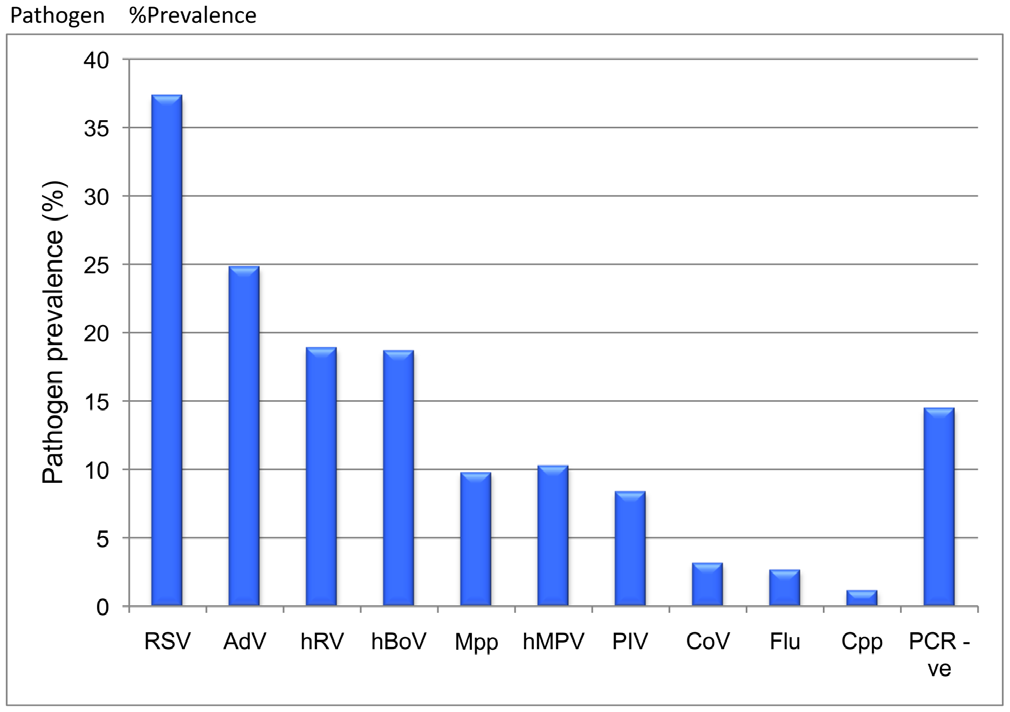
Pathogen prevalence (%) in nasopharyngeal aspirates from children less than five years with acute respiratory infection. Prevalence for viral/atypical pathogens and PCR negative samples is shown. Combined prevalence was greater than 100% because of co-infection. RSV, AdV, hRV and hBoV were most prevalent. (AdV, Adenoviruses; *Cpp*, *Chlamydia pneumoniae*; CoV, Coronaviruses; Flu, Influenza virus; hBoV, Bocavirus; hMPV, human Metapneumovirus; hRV, human Rhinovirus; *Mpp*, *Mycoplasma pneumoniae*; PIV, Parainfluenza virus; RSV, Respiratory syncytial virus).

Viral or atypical bacterial pathogens were detected in 348/407 (85·5%) NPAs. The most commonly identified pathogens were RSV (37·3% of patients), AdV (24·8%), hRV (18·9%), hBoV (18·7%), hMPV (10·3%) and *Mpp* (9·8%) [**Figure 1**]. Parainfluenza viruses were identified in 8·4% of samples with PIV3 representing the majority (4·9%). Coronaviruses were found in 3·2% of samples with OC43 being commonest (1·7%).

**Figure 2:**
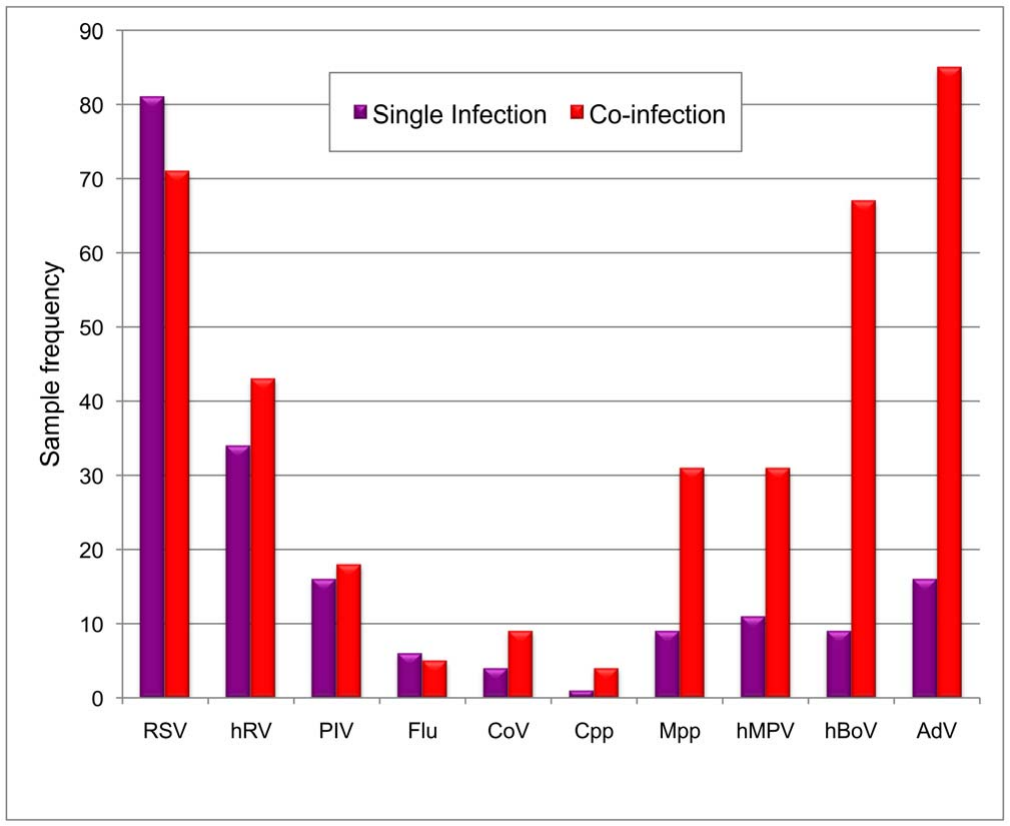
Pathogen frequency in singly infected (red) and co-infected (pink) nasopharyngeal aspirate samples. The frequency with which individual pathogens were identified in single and co-infections is shown. RSV was more likely to be identified as a single pathogen whereas AdV, hBoV, hMPV and *Mpp* were more likely to be identified in co-infections.

The commonest pathogens in single infections (n = 187; 45·9% total samples) were RSV (43·3%) and hRV (18·2%) with AdV and hBoV comprising a much smaller percentage (8·6% and 4·8% respectively) [**Figure 2**]. Co-infection with two or more pathogens was present in 161 samples (39·6%). Two pathogens were detected in 124 (30·5%) samples, three pathogens in 33 (8·1%) samples, four pathogens in 3 (0·7%) samples, and one sample contained five pathogens (a three month old girl with RSV, hMPV, hRV, *Mpp* and hBoV, who subsequently died of respiratory failure). The commonest pathogens in co-infected samples were AdV (52·8%), RSV (44·1%), hBoV (41·6%), hRV (26·7%) and *Mpp* (19·3%) with the most frequent combinations being RSV/hBoV (16·1% of co-infections) and RSV/AdV (11·2%).

### Disease Severity

**Figure 3:**
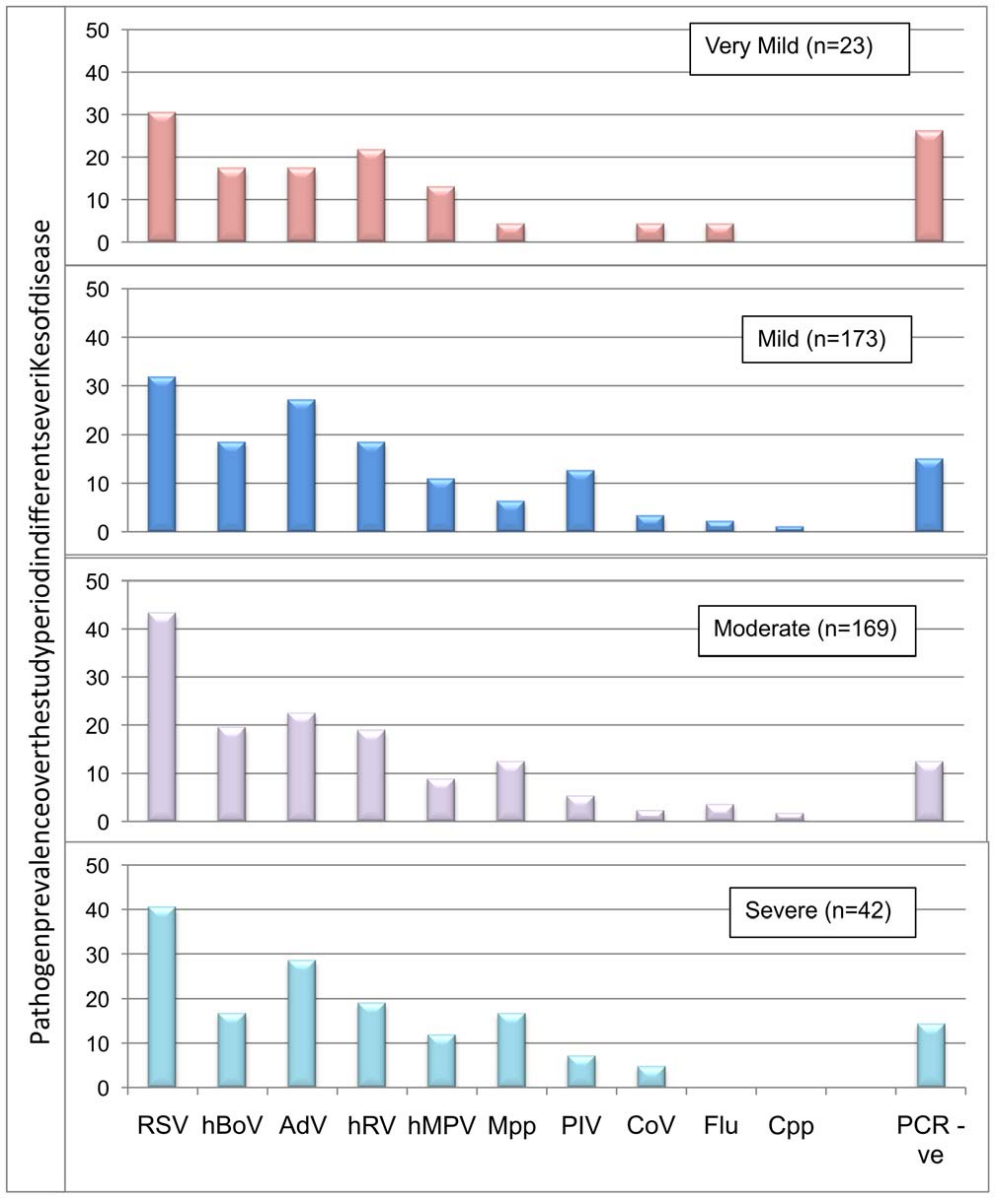
Relationship between pathogen prevalence and ARI disease severity. The prevalence with which each of the pathogens was identified in different severities of disease is shown. Most pathogens were represented within each of the disease severity groups, with prevalence being broadly similar. Children with RSV were more likely to have *moderate* than mild disease compared to children without RSV infection, whilst children with *Mpp* infection were more likely to have *moderate* or *severe* than *mild* disease (* p<0.05.). Cumulative prevalence in each of the disease severity groups was greater than 100% because of co-infection.

Overall, 5·7% (n = 23) children had *very mild* disease, 42·5% (173) *mild* disease, 41·5% (169) *moderate* disease and 10·3% (42) *severe* disease. Most pathogens were represented within each of the disease severity groups, with prevalence being broadly similar [**Figure 3**]. Children with RSV were more likely to have *moderate* than mild disease (43·2% vs. 31·8%; p = 0·019) compared to children without RSV infection, whilst children with *Mpp* infection were more likely to have *moderate* (12·4%) or *severe* (16·7%) than *mild* (6·4%) disease (*mild vs. moderate,* p = 0·04: *mild vs. severe*, p = 0·039).

Children with RSV and *Mpp* infection were more likely to be hospitalised than children without these pathogens. The proportion of children from whom no pathogen was detected was similar across the disease severity groups and between ambulatory and hospitalised patients. Likewise, no difference in disease severity was found in children in whom one, two, three or four pathogens were detected, even when AdV and/or hBoV were removed from the analysis.

### Clinical Manifestations

**Figure 4:**
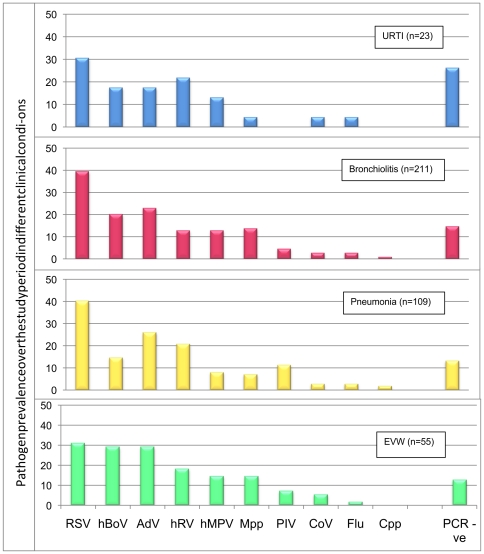
Relationship between pathogen prevalence and ARI clinical manifestation. Most pathogens were detected in each of the four major clinical manifestation groups with prevalence being broadly similar. Only *Mpp* infection was more commonly associated with pneumonia than bronchiolitis and hBoV was more commonly associated with EVW/asthma than bronchiolitis (* p<0.05). Cumulative pathogen prevalence in each of the clinical conditions was greater than 100% because of co-infection.

Overall, 5·7% (n = 23) children were discharged with a diagnosis of URTI, 51·8% (211) with bronchiolitis, 26·8% (109) with pneumonia, 13·5% (55) with EVW/asthma and 2·2% (9) with another diagnosis (whooping cough, pneumonia with pleural effusion and bronchiolitis obliterans). Most pathogens were detected in each of the four major clinical manifestation groups with prevalence being broadly similar [**Figure 4**]. Only *Mpp* infection was more commonly associated with pneumonia than bronchiolitis (13·8% of cases of pneumonia vs. 7·1% of cases of bronchiolitis; p = 0·04) and hBoV was more commonly associated with EVW/asthma than bronchiolitis (29·1% of EVW/asthma vs. 14·7% of bronchiolitis; p = 0·013). The proportion of patients from whom no pathogen was detected or in whom co-infection was found, was similar in all four major clinical manifestation groups.

Both RSV and *Mpp* were more commonly detected in hospitalised children with bronchiolitis than in ambulatory children with the same condition (*RSV,* hospitalised, 48%; ambulatory, 32%: p = 0·011; *Mpp*, hospitalised, 11%; ambulatory, 3%: p = 0·014). *Mpp* was detected in 17% of children hospitalised with pneumonia.

### Seasonal Distribution

**Figure 5:**
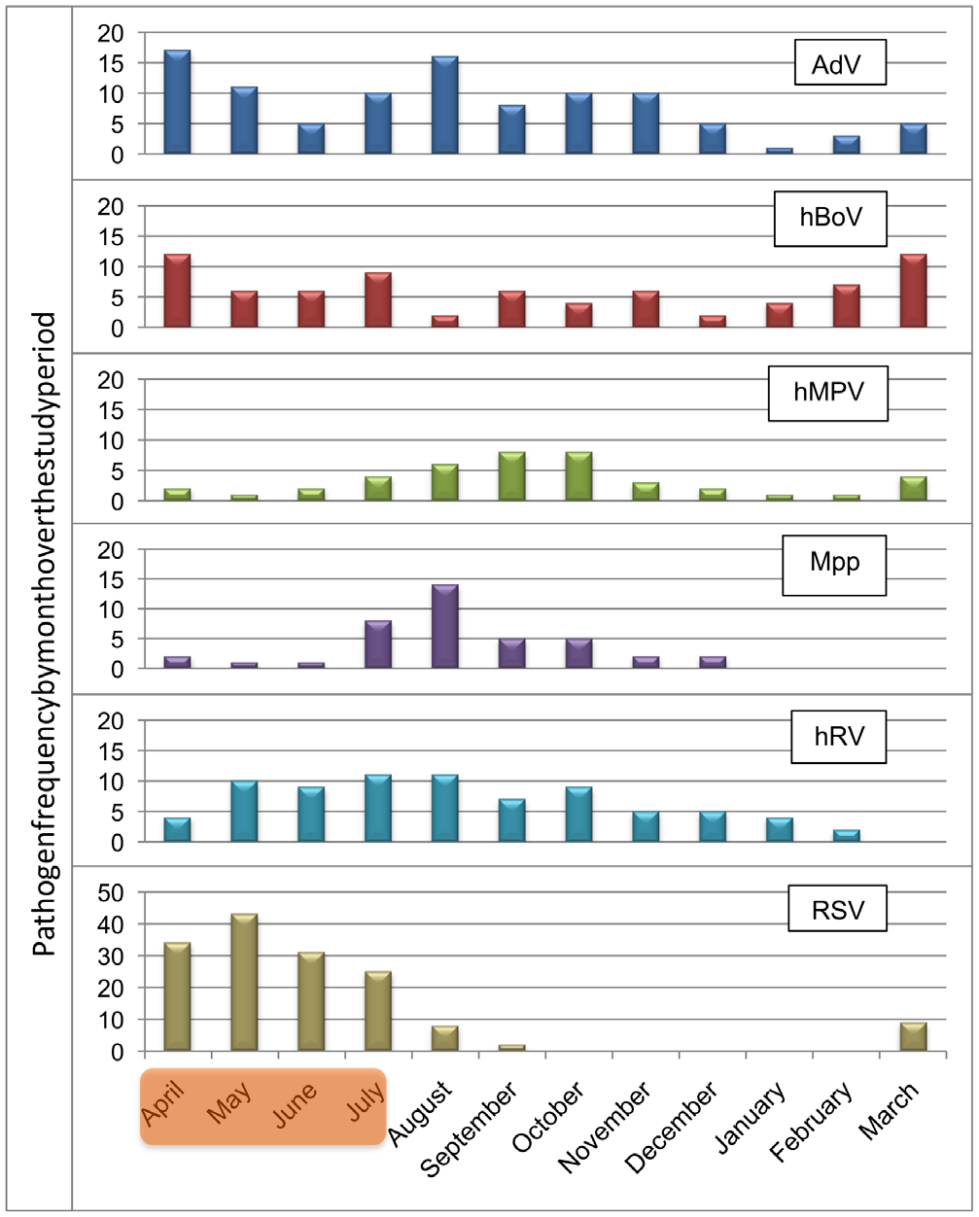
Monthly distribution of the most frequently detected pathogens in children with ARI. RSV, hMPV and *Mpp* exhibited strong seasonal patterns, with RSV cases peaking over the rainy season (Shaded area: April-July), and hMPV and *Mpp* peaking soon afterwards (August-October).

RSV, hMPV and *Mpp* exhibited strong seasonal patterns, with RSV cases peaking over the rainy season (April-July), and hMPV and *Mpp* peaking soon afterwards (August-October) [**Figure 5**]. AdV, hBoV and hRV were endemic throughout the year, although AdV activity did appear to decrease between December-March. In the year studied, different PIVs and CoVs circulated at different times of year.

## Discussion

This study describes for the first time the simultaneous detection of multiple respiratory pathogens in different severities and clinical manifestations of ARI in pre-school Brazilian children. Viral or atypical bacterial agents were detected in the majority of the children, with the commonest being RSV, AdV, hRV, hMPV, hBoV and *Mpp*. Most pathogens were detected in similar proportions across all severity categories, although RSV and *Mpp* were associated with more severe disease and were more commonly detected in children diagnosed with bronchiolitis and pneumonia. Two or more pathogens were detected in nearly 40% of the children, with some pathogens, such as AdV and hBoV, being frequently detected as co-infections whilst others, such as RSV and hRV, more commonly detected as single infections. The presence of co-infections was not associated with increased disease severity.

A viral or atypical bacterial pathogen was detected in 85·5% of the children, a detection rate particularly high compared to similar studies (35–78%).[12], [14], [19] There are a number of possible reasons for this. The study screened for more pathogens than previous studies and included hBoV, a virus particularly prevalent in pre-school children.[20] The NPA were collected prospectively by one person and processed rapidly, as opposed to other retrospective studies, which often include multiple specimen types collected by medical staff with varying training.[11], [21] A recent study of preschool children with ARI showed that pathogen detection by PCR was significantly higher in NPAs than nasal swabs.[22] Finally, the high rates of detection probably reflect the environment in which these children live.[4] Most came from low-income families living in urban slums, where overcrowding is common.

The high prevalence of AdV (24·7%) and hBoV (18·7%) co-infections could have been caused by asymptomatic persistence or prolonged nasopharyngeal shedding. Certainly AdV shedding in faeces can go on for many months following acute infection,[23] but while it is unknown whether this also occurs in the nasopharynx, no other study has found such high AdV detection rates.[11], [12], [13] Another possible explanation is that these findings may reflect living conditions, with AdV being a well-described cause of epidemic respiratory disease in crowded susceptible populations such as military trainees.[24] High rates of hBoV have been described [10], [25] with asymptomatic persistence in the upper airways for up to 2 months following an acute infection.[26] However, overrepresentation for one or both pathogens through contamination during sample collection or analysis cannot be excluded and further work would be needed to confirm these findings.

Conversely, detection of hRV was unexpectedly low. Competition within multiplex reactions can limit detection of low-level nucleic acid and may be particularly evident in co-infected samples.[18] Furthermore, the hRV primer set used in this study does not detect all hRV genotypes (e.g. hRV-72), which could lead to underrepresentation. Some members of hRV-C genogroup were detected and further work in this area is currently underway.

Very few studies have included the full spectrum of disease severity from mild to life-threatening disease. Over 80% of children in this study had mild or moderate disease, reflecting the majority of ARI seen in the A&E department. Interestingly most pathogens were detected across all disease severity categories, although RSV and *Mpp* were more frequently detected among children with severe disease, respectively confirming and highlighting the importance of these pathogens. RSV was also more likely to be detected in nasopharyngeal aspirates from hospitalised than ambulatory children with bronchiolitis. This finding is novel and potentially important for the assessment of RSV disease burden in the community but needs confirmation in other populations and settings.

Although detected in all of the major clinical manifestations of ARI, *Mpp* was more commonly associated with pneumonia. Perceived wisdom and published literature state that *Mpp* is a well-recognised cause of community-acquired pneumonia in older children and adults,[27] but a less common cause in preschool children, although few studies have examined prevalence in this age group.[12] Thus our findings that *Mpp* could be detected in approximately 10% of all children with ARI and 17% of hospitalised pneumonia cases is important and has potential implications for management, given that current treatment guidelines for pneumonia in this age group do not include macrolide antibiotics.[28] Interestingly, *Mpp* was more likely to be detected in hospitalised than ambulatory children with bronchiolitis, a finding which may in part reflect the difficulties in clinically distinguishing between pneumonia and bronchiolitis in young children.

In contrast with some previous studies,[15], [29] co-infection was not associated with disease severity. While this may be because we had smaller numbers of children in the very mild and severe categories, co-infection was consistently between 39–42% across all groups. Our study was also not powered to detect whether particular pathogen combinations were associated with more severe disease. Similarly, severe disease was no more or less common in those children in whom pathogens were not detected. In these children, one might speculate about possible infection with ‘typical’ bacteria such as Streptococcus and Haemophilus, although it is equally possible that viruses as yet unknown could have caused their symptoms.

Although this study examines the causes of ARI, we did not investigate pathogen prevalence in asymptomatic children from the same population. Van der Zalm *et al* recently showed that respiratory viruses (particularly RhV and CoVs) can be detected in 9–36% of respiratory samples from asymptomatic children of various ages[30], while a recent study by Berkley et al detected respiratory viruses in 28% of samples from well children[31]. Equally, while the study screened more pathogens than most published studies, some pathogens, such as *Bordetella pertussis* were not included. Other valid criticisms include that real-time PCR may be overly sensitive and that pathogen detection in the nasopharynx may not accurately reflect the situation in the lower respiratory tract. While neither criticism is limited to our study, we have tried to investigate the importance of our findings by putting them in the context of disease severity and clinical manifestation.

In summary, we have shown a very high prevalence of respiratory pathogens in preschool children in Brazil. Even without assessing the role played by ‘typical’ bacteria, we have highlighted the complexity of viral/atypical bacterial epidemiology in ARI in this age group and in this population and while simultaneous detection of pathogens using PCR will provide much useful information, it will also provide challenges of interpretation. Overcoming these challenges will be necessary to accurately direct resources and management, particularly in this age group where much of the morbidity and mortality associated with ARI lies.
